# Epidemiological Surveillance of Intestinal Parasites and Serological Analysis of *Toxoplasma gondii* in Captive Felids From Thailand Zoos

**DOI:** 10.1155/vmi/1596677

**Published:** 2025-07-07

**Authors:** Nuttanan Hongsrichan, Peerawich Donthaisong, Chavin Chaisongkram, Chatanun Eamudomkarn, Opal Pitaksakulrat, Kanda Ponsrila, Bandid Mangkit, Piangjai Chalermwong, Thitichai Jarudecha, Achirawit Surapinit, Thidarut Boonmars, Rucksak Rucksaken

**Affiliations:** ^1^Department of Parasitology, Faculty of Medicine, Khon Kaen University, Khon Kaen 40002, Thailand; ^2^Zoological Organization of Thailand, Khon Kaen Zoo, Khon Kaen 40280, Thailand; ^3^Department of Veterinary Technology, Faculty of Veterinary Technology, Kasetsart University, Bangkok 10900, Thailand; ^4^Department of Veterinary Nursing, Faculty of Veterinary Technology, Kasetsart University, Bangkok 10900, Thailand

**Keywords:** Felidae, intestinal parasite, *Toxoplasma gondii*, wild felids, zoo

## Abstract

**Background:** Wild felids have been in decline, which has been linked to health issues. Parasitic infections, including zoonotic ones, can contribute to the challenges faced regarding wildlife conservation efforts and may pose a risk to human health, particularly for people working at or visiting zoological parks. This study investigated the occurrence of parasitic infections in the Felidae within Thai zoo parks.

**Methods:** The fecal samples were collected from 93 Felidae individuals residing in four zoo parks. The parasitological technique involved sugar floatation, and a formalin-ethyl acetate concentration technique (FECT) was also employed. Additionally, a molecular technique was used to detect *Toxoplasma gondii* DNA in feces. For blood samples, 22 were tested for *T. gondii* DNA using the PCR technique. To detect *T. gondii* antibodies in serum, an indirect fluorescent antibody test (IFAT) was performed, and the result was confirmed using an enzyme-linked immunosorbent assay (ELISA).

**Results:** The prevalence of intestinal parasites in captive felids was 23.7%, with the highest rates observed for hookworms (8.6%), *Toxascaris leonina* (7.5%), coccidia (4.3%)*, Strongyloides* spp. (2.1%), and *Opisthorchis*-like egg (1.1%). Among the Felidae, lions and white lions (*Panthera leo*) exhibited the highest prevalence of parasitic infection, particularly *T. leonina*. Furthermore, a substantial percentage (63.6%) of the animals tested positive for *T. gondii* antibodies using both IFAT and ELISA.

**Conclusion:** These findings highlight the importance of addressing the circulation of parasites, such as *T*. *gondii*, hookworm, *T. leonina*, and coccidia in environments where animals and humans closely interact, such as wildlife zoos. Implementing preventative measures and adopting a one-health approach are strongly encouraged to control parasites and reduce the risk of infection for animals and humans.

## 1. Introduction

Populations of wild felids have declined due to health-related problems, such as nutritional deficiencies and infectious infections, including viruses, bacteria, and parasites [1]. Parasitic infections in captive wild Felidae are a major problem that has detrimental effects on the overall health and survival of individual animals. Common intestinal roundworms, such as *Toxocara* spp., *Toxascaris leonina*, *Ancylostoma* spp., *Strongyloides* spp., and lung fluke *Paragonimus westermani* and coccidia protozoa, such as *Neospora caninum* antibodies and *Toxoplasma gondii*, have been reported in zoo-kept Felidae worldwide [2–8]. In addition, wildlife infectious diseases caused by zoonotic parasites may represent a risk to human health, particularly animal keepers [9, 10], potentially serving as a transmission source for both humans and other animals [11]. Therefore, it is crucial to maintain up-to-date control measures for these parasitic infections.

In Thailand, there has been limited reporting regarding parasitic infection in animal zoos, especially for wild Felidae. A report in 1994 indicated a high prevalence of parasites in wild felids in Thailand [12]. A case study of leopard cats (*Prionailurus bengalensis*) reported that they were infected with *Hepatozoon* gamonts at the Khao Kheow Open Zoo, Chonburi, Thailand [13]. A case report has been published of a captive striped hyena (*Hyaena hyaena*) infected with *Armillifer armillatus* (visceral pentastomiasis) in the Chiang Mai Night Safari Park, Thailand [14]. However, it is necessary to conduct screening for *T. gondii* parasite antibodies in captive felids in zoos, and the data should be regularly updated [15]. Therefore, this study aimed to investigate parasitic infections in captive wild Felidae from several zoos in different regions of Thailand, using parasitological techniques, molecular technique for detection of *T. gondii* DNA in fecal samples, and serological approaches to identify *T. gondii* antibodies in sera. Understanding the health status of Felidae species in zoos, especially gastrointestinal parasitic infections, including *T. gondii*, is crucial for effectively managing and conserving wild Felidae and preventing the transmission of the parasite to other animals and potentially to humans [3], contributing to the well-being of captive felids and their long-term survival.

## 2. Materials and Methods

### 2.1. Study Area

**Figure 1:**
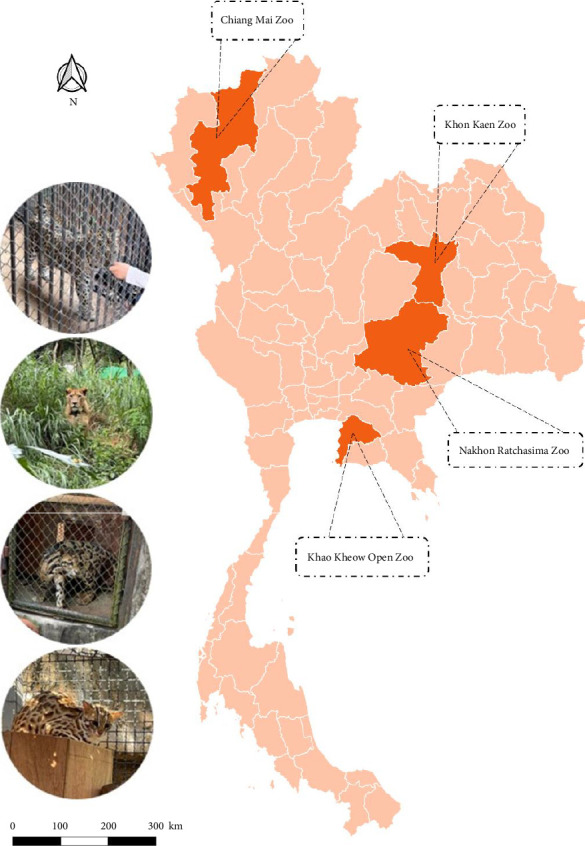
Sampling locations of zoos in different regions in Thailand. Map was generated using Quantum GIS (QGIS) software, Version 3.28.3.

Fecal samples were collected from April to October 2022, and blood samples were obtained from blood banks between 2021 and 2022 from four zoos across Thailand: the Chiang Mai Zoo, Khon Kaen Zoo, Nakhon Ratchasima Zoo, and Khao Kheow Open Zoo. The zoos are in different regions of Thailand, as shown in Figure 1.

### 2.2. Fecal Sample Collection

In total, 93 fecal samples were randomly collected from 14 Felidae species, with the number of samples per species shown in Table 1. Approximately 5–10 g of individual fresh fecal sample was collected in sterile, well-labeled zipper bags before cleaning the animal cages in the early morning by the zookeepers, and the portion that touched the ground was removed to avoid the chances of environmental contamination. Each fecal sample was labeled with the animal's name, common name, sex, and date of collection and then was immediately transported in an ice box to the laboratory of the Department of Parasitology, Khon Kaen University, Thailand. Each fecal sample was vigorously mixed and divided into three distinct experiments based separately on sugar flotation, a formalin-ethyl acetate concentration technique (FECT), and a molecular technique. For the sugar flotation and FECT methods, three parasitologists were involved in confirming the parasite eggs under the microscope.

### 2.3. Blood Sample Collection

The veterinary departments at the sampled zoos conduct annual health checkups on the Felidae family, collecting blood samples for both health assessments and storage in the blood bank. In total, 22 whole blood and serum samples were acquired from the same animals from which the fecal samples were collected. These samples were gathered from the four different zoos between 2021 and 2022. Whole blood samples were collected in EDTA tubes for polymerase chain reaction (PCR) analysis and stored at −20°C. The serum was obtained by clotting without anticoagulant, separated by centrifugation, and stored at −20°C until use in indirect fluorescent antibody test (IFAT) and enzyme-linked immunosorbent assay (ELISA) analyses.

### 2.4. Sugar Floatation

This technique was performed as described by Dryden et al. [16]. In brief, 2 g of feces were thoroughly mixed with 10 mL of a sugar flotation solution (modified Sheather's solution, specific gravity 1.27) and strained through a gauze. The sample was transferred to a 15-mL tube and then it was centrifuged at 1000 × *g* for 5 min. The filtrate was poured into a 15-mL tube, and sugar flotation solution was added up to 15 mL. The sample tube was kept for 15 min after placing a coverslip on top of each test tube, ensuring contact with the convex meniscus. Then, the coverslip was mounted on a glass slide and examined under a microscope.

### 2.5. FECT

A stool sample of approximately 3 g of fresh fecal sample was homogenized with 10 mL of normal saline filtered through a gauze and centrifuged at 1000 × *g* for 2 min. After removal of the supernatant, the sediment was resuspended in 7 mL of 10% buffered formalin and 3 mL of ethyl acetate. The sample was shaken vigorously and centrifuged at 1000 × *g* for 2 min. After centrifugation, the supernatant was discarded. The sediment was mixed and dropped on a glass slide and then observed under a microscope (Olympus, Japan).

### 2.6. IFAT for Detection of *T. gondii*

Antigen was generated by cultivating tachyzoites of the *T. gondii* RH strain, as described by Udonsom et al. [17]. On 6-well microscope slides (Electron Microscopy Sciences; USA), 15 μL of each Vero cell culture (1 × 10^6^ tachyzoites/mL) was spotted and air-dried overnight. All multiwell slides were fixed with acetone and kept at 20°C until utilized. Modifications were made to the IFAT techniques as described by Wiengcharoen et al. [18]. Each serum sample was diluted 1:16 with serum diluting buffer, pH 9.0 (VMRD, USA) for screening and doubled from 1:32 for positive sera. A 20-μL volume of each diluted sample was applied to 6-well microscope slides coated with *T. gondii* tachyzoites and incubated at 37°C for 1 h in a moisture-containing chamber. The slides were washed in FA rinse buffer (VMRD) for 3 and 10 min. Fluorescein isothiocyanate-labeled goat anti-cat IgG (KPL, USA) diluted to 1:400 was applied to each well on the slides prior to re-incubation at 37°C for 1 h in a humidified box. After a final washing of 10 min, the microscope slides were coated with coverslips and studied at 400× magnification under a fluorescent microscope (Olympus). Positive and negative samples were obtained from testing using the IFAT method and confirmation by the Parasitology Laboratory, Faculty of Veterinary Medicine, Kasetsart University. Positive and negative controls were processed alongside the samples at the same dilutions. All samples and controls for the slides were performed in duplicate. Positive results are characterized by bright, diffuse, or peripheral fluorescence of the tachyzoites, whereas a negative result is indicated by either no staining or only polar staining, as described by Smielewska-Los et al. [19] and Suwan et al. [20].

### 2.7. ELISA for Detection of *T. gondii*

All serum samples used in IFAT were analyzed with indirect ELISA to identify antibodies against *T. gondii* using the commercial kits: ID Screen Toxoplasmosis Indirect Multi-Species (TOXO-MS; ID.vet, Grabels, France), which utilized the P30 *T. gondii* tachyzoite surface protein as the antigen, with a multispecies horseradish peroxidase (HRP) conjugate as the secondary antibody and 3,3′,5,5′-tetramethylbenzidine (TMB) as the substrate. Tests were performed following the manufacturer's instructions. For each serum sample, a sample-to-positive ratio (S/P%) was calculated as S/P% = (OD sample - OD negative control/OD positive control - OD negative control) x 100, where OD represents the optical density of the sample, the positive control, or the negative control from the kits. Animals were classified by S/P% values, with S/P% ≤ 40% considered negative, 40% < S/P% < 50% inconclusive, and S/P% ≥ 50% positive, per the manufacturer's criteria.

### 2.8. Detection of *T. gondii* in Blood and Fecal Sample Using Conventional PCR

In total, 100 μL of whole blood and 200 mg of each fecal sample were extracted using a DNeasy blood and tissue kit (Qiagen, Germany) and a QIAamp PowerFecal DNA kit (Qiagen) according to the manufacturer's instruction. The extracted genomic DNA was determined for purity and concentration through 260/280 nm absorbance measurements using a NanoDrop spectrophotometer 2000 (Thermo Scientific, USA).

The primers specific for the *T. gondii* B1 gene (GenBank accession no. AF179871.1) were newly designed using the primer 3 software Version 4.1.0 (https://primer3.ut.ee). The forward and reverse primer sequences were 5′-GGTGTCGACAACAGAACAGC-3′ and 5′-GCCTCATTTCTGGGTCTACG-3′, respectively. The PCR product size of these primers was 179 bp. The PCR was carried out in a 12.5-μL volume, containing 1.0 μL of genomic DNA, 4.5 μL distilled water, 6.25 μL 2× DreamTaq Green PCR Master Mix, and 0.25 μL each of forward and reverse primer. The cycling profile consisted of an initial denaturation for 2 min at 95°C, 30 s at 95°C, 30 s at 61°C, 1 min at 72°C for 30 cycles, followed by a final extension at 72°C for 5 min. Sterile distilled water was used as a negative control, and the positive control used DNA from *T. gondii* tachyzoites RH strain obtained by DNA extraction, as describe previously [20]. The PCR products were mixed with 1-μL loading dye and were separated on an agarose gel containing 1.5% agarose using 1× Tris-borate-EDTA and observed under ultraviolet light.

### 2.9. Statistical Analysis

The statistical analysis was done using the Stata software package Version 17.0 (Stata Corporation, TX, USA). Univariable analysis was performed using McNemar's test for prevalence comparison between the sugar flotation and FECT techniques. Odds ratio (OR) of parasites infection in fecal samples between zoos and animals were analyzed by multivariable logistic regression and the confident interval (CI) of adjusted OR were 95%. Agreement between the IFAT and ELISA methods was assessed using Cohen's kappa coefficient, *κ*, where *κ* values from 0 to 0.20 indicate no agreement, 0.21 to 0.39 indicate minimal agreement, 0.40 to 0.59 indicate weak agreement, 0.60 to 0.79 indicate moderate agreement, 0.80 to 0.90 indicate strong agreement, and above 0.90 indicate almost perfect agreement [21]. Results were considered significantly different for *p* value < 0.05.

## 3. Results

**Figure 2:**
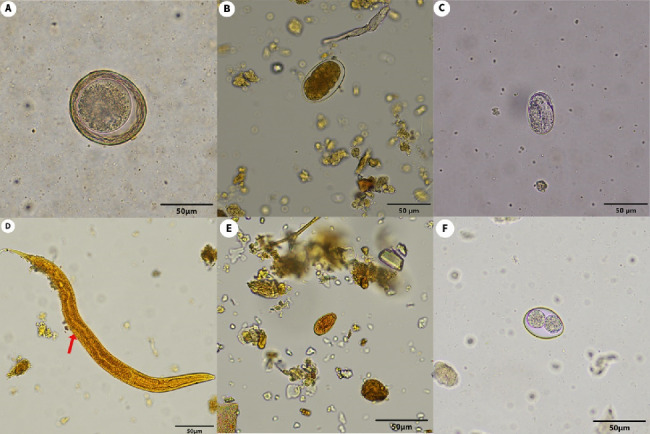
Parasites found in feces samples using formalin-ethyl acetate concentration technique and floatation techniques. *Toxascaris leonina* egg (A), hookworm egg (B), *Strongyloides* spp. egg (C), *Strongyloides* spp. rhabditiform larva (D), *Opisthorchis*-like egg (E), and Coccidia oocyst (F). The red arrow indicated prominent genital primordium.

In total, 93 fecal samples were collected from Felidae species in the zoos and were analyzed to detect intestinal parasites using two different techniques: sugar floatation and FECT. The highest prevalence of parasites (11/16, 68.75%) was observed in lions (*Panthera leo*), and the most frequently detected parasite in the fecal samples was hookworm (8/93, 8.6%), as shown in Table 1. The fecal examination results using sugar flotation and FECT are shown in Table 2 and Figure 2. The total prevalence of the intestinal parasite in the captive felids was 23.7% (22/93), with hookworm eggs being the most frequently found (8.6%), followed by *T. leonina* eggs (7.5%), coccidia oocysts (4.3%), *Strongyloides* spp. eggs and larvae (2.1%), and *Opisthorchis*-like eggs (1.1%), respectively. Only one sample of a white tiger (*Panthera tigris*) was coinfected with the helminth *T. leonina* and protozoa coccidia. In addition, *T. gondii* oocysts were not detected by either of the two fecal examination methods. There was a significant difference between the prevalence rates of hookworms (*p*=0.0078) in the zoo Felidae based on sugar floatation and FECT for detection (Table 2).

**Figure 3:**
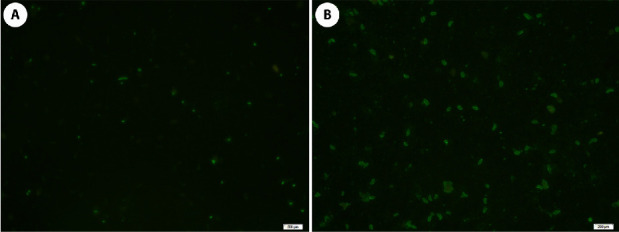
Indirect fluorescent antibody test (IFAT) for toxoplasmosis detection in serum samples. Negative control (A) and positive IFAT reaction using *T. gondii* RH strain as antigen (B) under 400× magnification.

*T. gondii* antibodies were detected based on the IFAT using the RH strain tachyzoite as the antigen. The positive cases showed diffuse fluorescent staining of tachyzoite cells, while the negative samples had no fluorescent staining or apical (polar) staining, as shown in Figure 3. Seroprevalence from the ELISA test based on the P30 *T. gondii* tachyzoite surface protein as the antigen showed 100% concordance with the IFAT result (Table 3). The correlation indicated perfect agreement between IFAT and ELISA tests (*κ* = 1). Using both methods, *T. gondii* antibodies were detected in 63.6% (14/22) of the captive felids from all the zoos. Based on the IFAT test titer, all positive sera were detected at titer of 1:16, with 63.6% (14/22) samples positive with a titer of 1:32, while 54.5% (12/22) samples were positive at a titer of 1: 64, and only 22.7% (5/22) samples were positive with a titer of 1:128 (Table 4). The infection rates were 100% for *T. gondii*, in the fishing cat (*Prionailurus viverrinus*) (2/2), Siberian tiger (*Panthera tigris altaica*) (2/2), white tiger (*P. tigris*) (1/1), and leopard (*Panthera pardus*) (1/1). In contrast, the Indochinese tiger (*Panthera tigris corbetti*) had a 75% (3/4) infection rate, while the lion (*P. leo*) had a 66.7% (2/3) infection rate. The clouded leopard (*Neofelis nebulosa*) had a 42.9% (3/7) *T. gondii* infection rate, while none of the samples from the jaguars (*Panthera onca*) (0/2) showed any antibodies to *T. gondii* (Table 3). In addition, we attempted to identify *T. gondii* in blood and stool samples using molecular techniques. However, none of the samples revealed the presence of the parasites. After being adjusted, there was no difference in the OR of parasite infection in wild Felidae between zoos and animal category (Table 5).

## 4. Discussion

Captive populations of wild felids can play a major role in the ecology and transmission of parasites [22]. The current study investigated the presence of intestinal parasites and *T. gondii* infections within four Thai zoos across different regions of Thailand (North, Northeast, and East). The intestinal parasites that were most frequently found were hookworms, *T. leonina*, coccidia, *Strongyloides* spp., and *Opisthorchis*-like egg, respectively. The findings demonstrated that each parasitological technique (floatation or FECT) produced distinct results for identifying each parasite, suggesting that both techniques should be used to identify intestinal parasitic infection. In this study, FECT performed better in overall detection of intestinal parasites in zoo Felidae, and *Opisthorchis*-like egg is only detectable using this technique. This result is consistent with the previous study in cats in Thailand [23]. The sugar flotation method (specific gravity 1.27 with 0.05% Tween-80) is considered efficient for floating helminth eggs [24]. However, this study did not detect hookworm eggs using this technique. Flotation methods can yield false negatives in hookworm egg detection due to factors such as egg adhesion to instrument surfaces, interference from fats in fecal samples, and the method's detection limits. This suggests that using multiple diagnostic methods may be necessary for optimal detection results [25]. In addition, the results suggested that the frequency of zoonotic protozoans and gastrointestinal helminths in zoo Felidae analyzed in Thailand was low. However, these animals may serve as a potential reservoir and transmit these parasites between animals and humans.

*T. leonina* is a prevalent parasitic worm in domestic cats because it is easily acquired through ingestion of infective eggs [26]. The importance of *T. leonina* as a potential zoonotic threat is being increasingly recognized due to the intimate bonds between humans and pets, as well as the heightened human–wildlife interactions observed in zoo settings [27]. Given the substantial population of stray dogs and cats in Thailand, along with additional paratenic hosts, such as rodents, these stray animals could contribute to the transmission of parasites to captive felid species within zoo settings. *Strongyloides* spp. and hookworm share a common method of transmission, and the third-stage infective larvae (L3) of these two parasites can penetrate the skin of animals. Thus, transmission might occur through skin penetration, especially during the rainy season. Both *Strongyloides* spp. and hookworms have zoonotic capabilities and are prevalent throughout Thailand [28, 29]. The role of stray dogs and cats in transmitting *Strongyloides* spp. and hookworms into the zoo environment deserves further investigation. Although efforts are made to reduce fecal contamination of the soil, some contamination may still occur, allowing for the potential detection of *Strongyloides* larvae.

The current study was the first report of a liver fluke, *Opisthorchis*-like egg infection, in zoo Felidae in Thailand. However, considering that *Opisthorchis viverrini* is endemic to North and Northeast Thailand [30], detecting *Opisthorchis*-like eggs in an Asiatic golden cat in the Chiang Mai Zoo was not unexpected. Notably, *O. viverrini* is the most prevalent species in Thailand and the Mekong Basin of Southeast Asia [30]. This parasite can cause opisthorchiasis-related cancers, particularly cholangiocarcinoma, impacting both humans and animals. The current findings of *Opisthorchis*-like eggs highlight that liver fluke transmission among humans, domestic animals, and zoo animals remains a concern in endemic areas.

The current research used the IFAT and ELISA to identify antibodies against *T. gondii*. This intracellular protozoan parasite has a complex life cycle that involves felids as definitive hosts [31]. Toxoplasmosis can affect various organs and systems in infected animals [32]. In many cases, felids infected with *T. gondii* do not exhibit any visible symptoms or only experience mild illness that goes undetected [7]. Toxoplasmosis is one of the most common parasitic infections worldwide, with zoonotic potential. Its high prevalence has been reported in both human and animal populations [32]. The estimated seroprevalence in wild felids was reported as 67% in Asia and more than 57% worldwide [33]. While ELISA is the most widely used test [20, 34, 35], another commonly used serological test for toxoplasmosis is the IFAT [2, 20]. Other serological tests, such as the latex agglutination test, may also be used to detect *T. gondii* antibodies [15]. This study used both IFAT and ELISA to determine the seroprevalence of *T. gondii*. Results from IFAT showed complete agreement with ELISA, revealing a remarkably high prevalence of *T. gondii* antibodies in zoo Felidae at 63.6%, which was higher than a study in 2006 that reported *T. gondii* antibodies in 42.8% of wild felids using the Sabin–Feldman dye test [36] and a seroprevalence of 15.4% in captive wild felids using a commercial latex agglutination test kit [15]. Furthermore, the prevalence was relatively high compared to the research results on stray cats (4.8%) [37] and adopted cats that roamed the zoo (10.53%) [38]. In China, cats and other felids from zoos have a very high seroprevalence of *T. gondii* infection [39, 40], similar to the high seroprevalence (47.2%) found in free-ranging cats from Malaysia [41]. However, the occurrence of *T. gondii* in wild felids differs with the country. A definitive host only spreads environmentally resistant oocysts during the initial weeks of infection, which was probably why our PCR protocol did not detect *T. gondii* DNA in the blood and fecal samples of the positive cases.

Wildlife is an important factor in conservation efforts because they face threats, with some species being critically endangered. The current study contributed to increasing the awareness of wild felid health and should support the establishment of a policy for the prevention, control, and eradication of intestinal parasites, including *T. gondii* in zoo felids across Thailand. Notably, the identified parasites included hookworm, *T. leonina*, coccidia, *Strongyloides* spp., and *Opisthorchis*-like eggs. Therefore, particular emphasis should be placed on preventing the transmission of these parasites to humans.

## 5. Conclusions

This research discovered that wild felids were harboring numerous zoonotic helminth parasites. Additionally, a notably high seroprevalence rate of *T. gondii* antibodies was identified in the sampled wild felids residing in the four different zoos, underscoring the importance of implementing effective measures to control the transmission of parasites between humans and zoo animals.

## Figures and Tables

**Table 1:** Prevalence of parasites infection in fecal samples of wild Felidae according to host species.

Animal	Scientific name	Prevalence (%)						
		*n*	*T. leonina* (egg)	*Strongyloides* spp. (egg and larva)	Hookworm (egg)	*Opisthorchis*-like (egg)	Coccidia (oocyst)	Total positive
Ocelot	*Leopardus pardalis*	1	0	0	1	0	0	1
Leopard	*Prionailurus bengalensis*	12	0	1	0	0	1	2
Fishing Cat	*Prionailurus viverrinus*	10	0	0	1	0	0	1
Jungle Cat	*Felis chaus fulvidina*	1	0	0	0	0	0	0
Clouded leopard	*Neofelis nebulosa nebulosa*	28	0	0	4	0	0	4
Panther	*Panthera pardus*	1	0	0	0	0	0	0
Puma	*Puma concolor*	1	0	0	0	0	0	0
Indochinese leopard	*Panthera pardus delacourii*	1	0	0	0	0	0	0
Jaguar	*Panthera onca*	3	0	0	0	0	0	0
Black jaguar	*Panthera onca*	2	0	0	0	0	0	0
Indochinese tiger	*Panthera tigris corbetti*	11	0	0	1	0	1	2
White tiger	*Panthera tigris*	2	0	0	0	0	0	0
Siberian tiger	*Panthera tigris altaica*	2	0	0	0	0	0	0
Lion	*Panthera leo*	5	3	0	0	0	2	5
White lion	*Panthera leo*	9	3	1	1	0	0	5
African lion	*Panthera leo*	2	1	0	0	0	0	1
Asiatic golden Cat	*Catopuma temminckii*	2	0	0	0	1	0	1
Total		93	7 (7.5%)	2 (2.1%)	8 (8.6%)	1 (1.1%)	4 (4.3%)	22 (23.7%)

Abbreviation: *T*. *leonina*, *Toxascaris leonina*.

**Table 2:** Prevalence of parasite infections in wild Felidae using sugar flotation and formalin-ethyl acetate concentration techniques.

Parasites	No. of positive (%prevalence)^∗^	No. of positive sample parasitological technique		*p* value
		Sugar flotation	FECT	
*Toxascaris leonina* egg	7	7	5	0.500
*Strongyloides* spp. egg and larva	2	2	0	0.500
Hookworms egg	8	0	8	0.0078^∗∗^
*Opisthorchis*-like egg	1	0	1	1.000
Coccidia oocyst	4	3	1	0.500
Total (*n* = 93)	22 (23.7)	12 (12.9%)	15 (16.1%)	0.1094

*Note:* FECT, formalin-ethyl acetate concentration technique.

^∗^No. of sample that was positive by at least one parasitological technique.

^∗∗^ *p* values < 0.05 were considered significant, statistical analysis by McNemar's test.

**Table 3:** The detection of *T. gondii* using the immunofluorescence antibody technique (IFAT) and enzyme-linked immunosorbent assay (ELISA).

Species	Total	IFAT + (%)	IFAT - (%)	ELISA + (%)	ELISA - (%)
*Panthera tigris altaica*	2	2 (100%)	0 (0%)	2 (100%)	0 (0%)
*Panthera leo*	3	2 (66.7%)	1 (33.3%)	2 (66.7%)	1 (33.3%)
*Panthera tigris*	1	1 (100%)	0 (0%)	1 (100%)	0 (0%)
*Panthera onca*	2	0 (0%)	2 (100%)	0 (0%)	2 (100%)
*Prionailurus viverrinus*	2	2 (100%)	0 (0%)	2 (100%)	0 (0%)
*Panthera tigris corbetti*	4	3 (75%)	1 (25%)	3 (75%)	1 (25%)
*Neofelis nebulosa*	7	3 (42.9%)	4 (58.1%)	3 (42.9%)	4 (58.1%)
*Panthera pardus*	1	1 (100%)	0 (0%)	1 (100%)	0 (0%)
Total	22	14 (63.6%)	8 (36.4%)	14 (63.6%)	8 (36.4%)

**Table 4:** Serial dilutions for *T. gondii* detection using immunofluorescence antibody technique (IFAT).

Serial dilution			
Dilution	Number of positive	Number of negative	Total
1:16	14	8	22
1:32	14	8	22
1:64	12	10	22
1:128	5	17	22
1:256	0	22	22

**Table 5:** Odds ratio of parasite infection in fecal samples of wild Felidae between zoos and animal.

Factor	Odds ratio	95% CI	*p* value
Zoo			
Khao Kheow Open Zoo	1.0	(Reference)	
Khon Kaen	1.30	0.10–16.80	0.843
Nakhon Ratchasima	0.86	0.04–17.64	0.921
Chiang Mai	2.73	0.30–24.86	0.374
Animal			
Clouded leopard	1.0	(Reference)	
Leopard	0.44	0.02–9.60	0.601
Lion	5.81	0.41–82.38	0.193
White lion	1.77	0.12–25.62	0.674
Indochinese tiger	0.48	0.03–6.62	0.583
Fishing cat	0.55	0.04–7.55	0.657
Asiatic golden cat	4.11	0.09–179.12	0.462
African lion	7.34	0.11–482.70	0.351

*Note:* Odds ratios were performed using multivariable logistic regression analysis, and *p* values < 0.05 were considered significant. White jiger, Jaguar, Siberian tiger, jungle cat, Indochinese leopard, puma, black jaguar, ocelot, and panther were not applicable.

## Data Availability

The data that support the findings of this study are available from the corresponding author upon reasonable request.

## Nomenclature

*T. leonina*: *Toxascaris leonina*
*T. gondii*: *Toxoplasma gondii*
spp.: Species
SF: Sugar floatation
FECT: Formalin-ethyl acetate concentration technique
ELISA: Enzyme-linked immunosorbent assay.
